# Supplementary material for: Preclinical Synergistic Combination Therapy of Lurbinectedin with Irinotecan and 5-Fluorouracil in Pancreatic Cancer
Source: Curr Oncol. 2023 Oct 31;30(11):9611–26. doi: 10.3390/curroncol30110696 (PMC10670398; doi:10.3390/curroncol30110696)

This is the original Western Image for Appendix A, which was cropped to generate Figure 4B and Figure 4D.

There are 12 lanes in each sample: 6 lanes, then ladder, then another 6 lanes. It is organized as follows:

							¦						
Lurbinectedin	–	+	–	–	+	–	¦	–	+	–	–	+	–
Irinotecan	–	–	+	–	+	+	¦	–	–	+	–	+	+
ONC212	–	–	–	+	–	+	¦	–	–	–	+	–	+

ONC212 is not a component of this paper, and thus was cropped out to generate Figure 4.

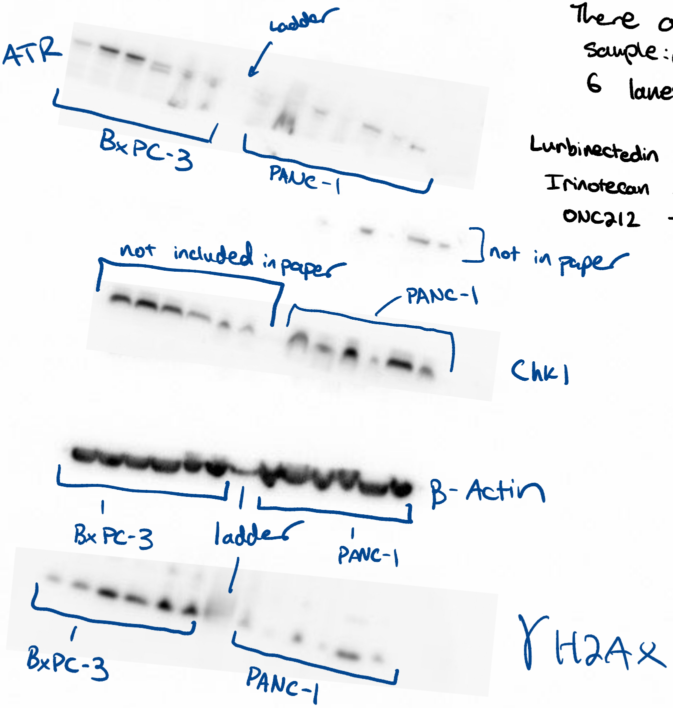

These are the original images for the western blots used to make Figure 4A and Figure 4C.

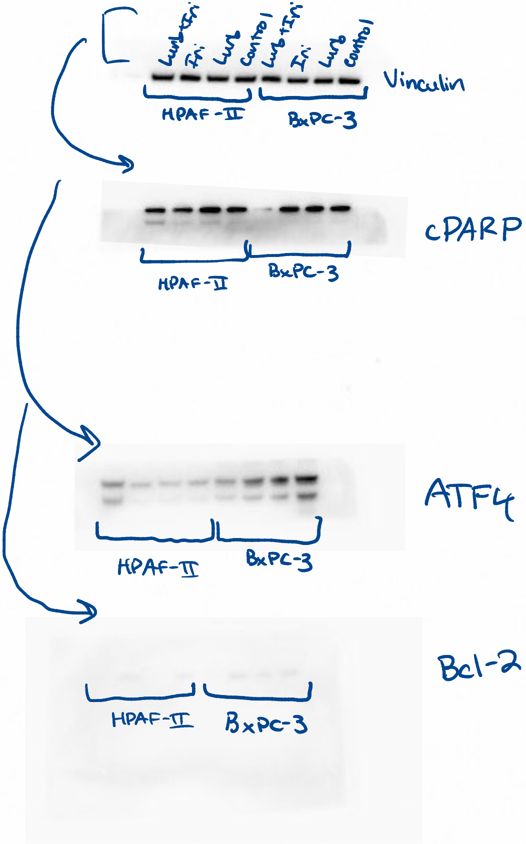

Supplement: Supplementary file 1 [file curroncol-30-00696-s001.zip › Western Blot Fully Labeled Original Image for CO.pdf]
