# Supplementary figures and images for: Preclinical Synergistic Combination Therapy of Lurbinectedin with Irinotecan and 5-Fluorouracil in Pancreatic Cancer
Source: Curr Oncol. 2023 Oct 31;30(11):9611–26. doi: 10.3390/curroncol30110696 (PMC10670398; doi:10.3390/curroncol30110696)

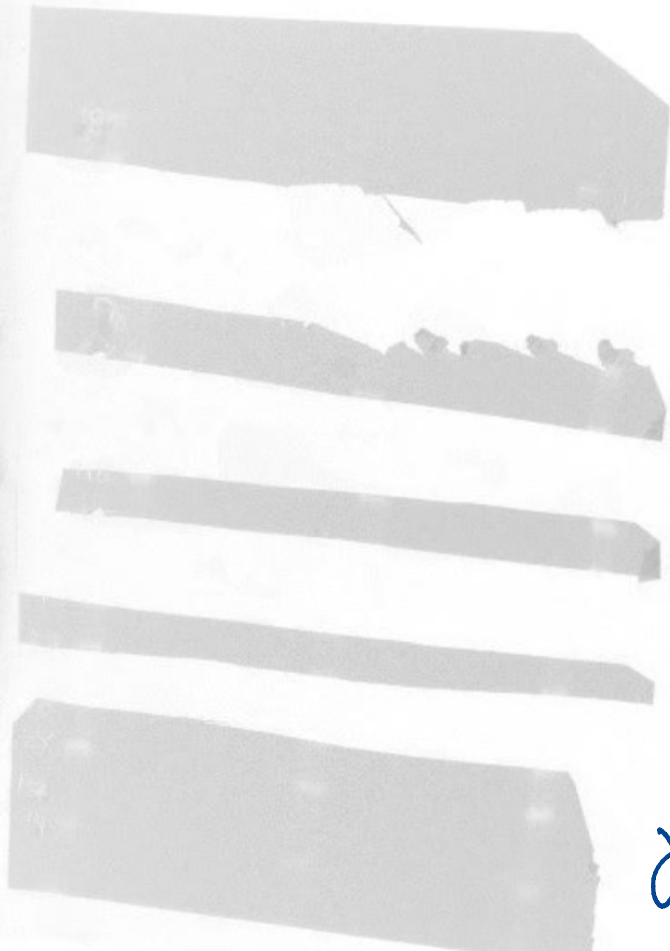

ATR

N/A

CHK-1

Actin

$\gamma$ H2AX

Gel 1

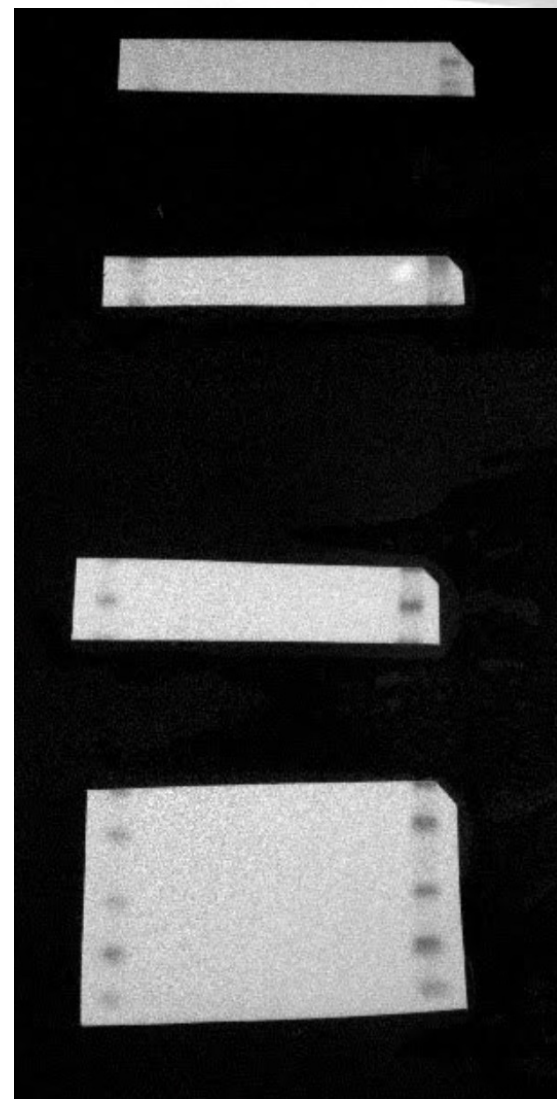

Vinculin

cPARP

ATF-4

Bcl-2

Gel 2

Supplement: Supplementary file 1 [file curroncol-30-00696-s001.zip › Raw Western Blot Images.pdf]
